# RETRACTED: Nazir et al. Curative Effect of Catechin Isolated from *Elaeagnus Umbellata* Thunb. Berries for Diabetes and Related Complications in Streptozotocin-Induced Diabetic Rats Model. *Molecules* 2021, *26*, 137

**DOI:** 10.3390/molecules31071183

**Published:** 2026-04-02

**Authors:** Nausheen Nazir, Muhammad Zahoor, Riaz Ullah, Essan Ezzeldin, Gamal A. E. Mostafa

**Affiliations:** 1 Department of Biochemistry, University of Malakand, Chakdara Dir (L), Khyber Pakhtunkhwa 18800, Pakistan; mohammadzahoorus@yahoo.com; 2 Department of Pharmacognosy, College of Pharmacy, King Saud University, Riyadh 11451, Saudi Arabia; rullah@ksu.edu.sa; 3 Department of Pharmaceutical Chemistry, College of Pharmacy, King Saud University, Riyadh 11451, Saudi Arabia; esali@ksu.edu.sa (E.E.); gmostafa@ksu.edu.sa (G.A.E.M.); 4 Micro-Analytical Laboratory, Applied Organic Chemistry Department, National Research Center, Dokki, Cairo 12622, Egypt

The journal retracts the article “Curative effect of catechin isolated from *Elaeagnus umbellata* Thunb. Berries for diabetes and related complications in Streptozotocin-induced diabetic rats model” [1] cited above.

Following publication, concerns were brought to the attention of the Editorial Office regarding the integrity of a number of panels within Figure 6 presented in this article [1].

Adhering to our standard procedure, an investigation was conducted by the Editorial Office and Editorial Board which identified evidence of inappropriate editing and duplication in panels within Figure 6 of this article [1], as well as partial duplication between this figure and one from another publication [2], authored by a partially overlapping group and describing different experimental conditions. While the authors collaborated within this process, the raw material requested for evaluation by the Editorial Board could not be provided. Consequently, the Editorial Board has lost confidence in the reliability of the findings and decided to retract this publication [1], as per MDPI’s retraction policy (https://www.mdpi.com/ethics#_bookmark30).

This retraction was approved by the Editor-in-Chief of the journal *Molecules.*

The authors did not agree to this retraction.
